# Technogenic Magnetic Particles in Alkaline Dusts from Power and Cement Plants

**DOI:** 10.1007/s11270-012-1389-9

**Published:** 2012-11-27

**Authors:** Tadeusz Magiera, Beata Gołuchowska, Mariola Jabłońska

**Affiliations:** 1 Department of Land Protection, University of Opole, Opole, Poland; 2 Institute of Environmental Engineering, Polish Academy of Sciences, Zabrze, Poland; 3 Department of Geochemistry, Mineralogy and Petrology, Faculty of Earth Sciences, University of Silesia, Sosnowiec, Poland

**Keywords:** Alkaline dusts, Magnetic susceptibility, Technogenic magnetic particles, Iron mineralogy

## Abstract

During this study, we investigated the mineralogical characterization of technogenic magnetic particles (TMPs) contained in alkaline industrial dust and fly ash emitted by coal burning power plants and cement plants. The reaction of tested dust samples varied between values of pH 8 and pH 12. Their magnetic properties were characterized by measurement of magnetic susceptibility (*χ*), frequency dependence of magnetic susceptibility (χ_fd_), and temperature dependence of magnetic susceptibility. Mineralogical and geochemical analyses included scanning electron microscopy with energy dispersive spectroscopy, microprobe analysis and X-ray diffraction. The TMPs in fly ash from hard coal combustion have the form of typical magnetic spherules with a smooth or corrugated surface as well as a skeletal morphology, composed of iron oxides (magnetite, maghemite, and magnesioferrite) that occurred in the form of incrustation on the surface of mullite, amorphous silica, or aluminosilicate particles. The TMPs observed in fly ash from lignite combustion have a similar morphological form but a different mineralogical composition. Instead of magnetite and magnesioferrite, maghemite and hematite with lower *χ* values were the prevailing magnetic minerals, which explains the much lower magnetic susceptibility of this kind of ash in comparison with the ash from hard coal combustion, and probably results from the lower temperature of lignite combustion. Morphology and mineralogical composition of TMPs in cement dust is more diverse. The magnetic fraction of cement dust occurs mostly in the form of angular and octahedral grains of a significantly finer granulation (<20 μm); however, spherules are also present. A very characteristic magnetic form for cement dust is calcium ferrite (CaFe_3_O_5_). The greatest impact on the magnetic susceptibility of cement dust results from iron-bearing additives (often waste materials from other branches of industry), which should be considered the most dangerous to the environment. Stoichiometric analysis of micro-particles confirmed the presence of heavy metals such as Pb, Mn, Cd, and Zn connected with TMPs, which are carriers of magnetic signals in atmospheric dust. Therefore, in some cases, their presence in topsoil when detected by magnetic measurement can be treated as an indicator of inorganic soil contamination.

## Introduction

The wide use of coal as an energy source, combined with the intensive development of many branches of industry, including chemical, ceramic, and cement production at the beginning of the twentieth century, has resulted in the emission into the atmosphere of extremely large amounts of industrial dust and fly ash. This dust usually has an alkaline reaction (pH > 7.2) and contains a significant amount of trace elements, constituting a potential threat to the natural environment and living organisms. Dust particles deposited on the soil surface, in contact with the acidic soil environment, especially in forest areas, may cause the release of potentially toxic trace elements that create a threat to the environment (Strzyszcz and Magiera 1998; Klose et al. 2001; Polat et al. 2004).

Alkaline dust is also deposited directly on the surface of plants. Taking into account that the size of the stoma in stomata apparatus varies from 8 to 10 μm, dust particles with a diameter similar to that of the stoma can block its aperture. However, smaller particles can penetrate directly into leaf tissue. Despite the fact that the leaves of most plants are protected by cuticle, this protective layer can be dissolved by alkaline dust, especially cement and lime dust which contains large amounts of calcium oxide. It has been proven that pine bark, with a pH of between 3 and 4, can be substantially damaged during contact with aggressive alkaline dust of a pH higher than 10 (Farmer 1993; Migaszewski et al. 2001).

Much more hazardous to the environment are heavy metals contained in alkaline dust. Heavy metals in fly ash originate from the mineral content of coal and—during its combustion—move from the furnace, through the slag traps, de-dusting devices, and up the chimney. The presence of heavy metals (Be, Cr, Zn, Co, Mo, Ni, Pb, and Ti) in various grain fractions of fly ash from Polish power plants, and the close relationship between metal content in ash and the specific surface area of its particles, has been frequently observed (Konieczyński and Żeliński 2009). A very high risk to the environment is posed by fine dust fraction. Fe-rich, magnetic particles found in atmospheric dust in Rome were mostly 0.1–5 μm in size (Sagnotti et al. 2009). This fraction is characterized by the greatest content of metals and the least effective retention by electrostatic precipitators. Geochemical analysis has shown that the content of trace elements such as Zn, Ga, Cd, Cu, Mo, Pb, and Tl, known as easily volatile, increases with the increase in the degree of fineness of ash particles. This occurs as a result of vaporization of these elements during coal combustion, followed by deposition and surface condensation on ash grains after lowering the ambient temperature (Konieczyński et al. 2007; Konieczyński and Stec 2009).

The degree of potential toxicity of heavy metals depends on the form of binding with the mineral phase. Research by Hullet et al. (1980), and also later other authors (Hansen et al. 1981; Vassilev et al. 2001, 2004) show that most of the elements belonging to the first and second transition series, such as Pb, Zn, Cd, V, Cr, Co, Ni, and Cu, occurring in coal mainly in the form of sulfides, are bound with magnetic minerals in fly ash. The crystallographic structure of magnetite and various ferrites formed at high temperatures allows for the introduction of numerous elements into fly ash, which can be potentially hazardous for plants, animals, and humans in the soil environment. Geochemical studies of fly ash have proven that heavy metals can also be adsorbed on a particle’s surface (Vaughan et al. 2002; Giere and Querol 2010). In particular, the finest fraction of fly ash, with a highly developed specific surface area, may be enriched in such metals as Cr, Mn, Pb, V, and Zn (Keyser et al. 1978). Heavy metals that are only connected to the surface of ash particles by adsorption forces are easily activated in the soil under the influence of organic acids and pose a serious hazard to the biological environment. The progressive acidification of soils associated with emissions of SO_2_ and then deposition in the form of acid rain accelerates the release of heavy metals.

By the 1980s, many researchers had already noted the relationship between increase in magnetic susceptibility and the content of heavy metals in many environments. Hunt et al. (1984) show a linear correlation between the content of magnetic particles in urban dust and the content of such metals as Pb, Cu, Zn, and Cd. A similar correlation between magnetic susceptibility and the content of Cu, Fe, Pb, and Zn in atmospheric dust is observed by Beckwith et al. (1986). The presence of magnetic particles was also found in cement dust (Strzyszcz 1995; Gołuchowska 2001). In this case, the main source of magnetic particles and associated heavy metals are additives and fuels used in the process of clinker burning. These additives are often waste materials from other branches of industry, such as metallurgical waste, cinder pyrites, fly ash etc.

The research presented in this work aimed at carrying out a detailed description of mineralogical characteristics and morphology of the mineral particles of alkaline dust from power stations and cement plants of southern Poland, with special regard to the magnetic phase of this dust as the main carrier of heavy metals. This phase, easily detectable by geophysical measurements, can be used as an indicator supporting quantitative evaluation of the content of potentially toxic metals in the environment.

## Materials and Methods

In this study, 15 samples of fly ash were examined; of which ten came from nine Polish power plants burning hard coal, and five from lignite burning power plants. In addition, 12 samples of cement and lime dust arising from plants located in the province of Opole (Southern Poland) were tested. Fly ash was collected from different areas of electrofilters. Cement dust was taken both from electrofilters and from the dustfall in the vicinity of cement plants.

The first step of the study was to take measurements of low-frequency magnetic susceptibility (κ) in the laboratory using an MS2 “Bartington” magnetic susceptibility meter and MS2B sensor. The result of the measurements obtained for a dust sample of a certain density was converted to mass magnetic susceptibility (*χ*) and expressed as cubic meters per kilogram (Thompson and Oldfield 1986).

To determine frequency-dependent magnetic susceptibility (*χ*_fd_), additional measurements at high frequency (4,700 Hz) were carried out. Frequency-dependent magnetic susceptibility is a parameter that helps to determine the presence of superparamagnetic fraction in the tested sample, i.e., magnetic particles <0.02 μm. The *χ*_fd_ parameter is expressed in percentages and calculated by the formula:

$$ {\chi_{fd }}={\chi_{\mathrm{LF}}}-{\chi_{\mathrm{HF}}}/{\chi_{\mathrm{LF}}}\cdot 100\% $$

where:

*χ*_fd_: Frequency-dependent magnetic susceptibility
*χ*_LF_: Magnetic susceptibility measured in low magnetic field at a frequency of 470 Hz
*χ*_HF_: Magnetic susceptibility measured in low magnetic field at a frequency of 4,700 Hz.

In the second stage, pH measurements of the tested dust samples were undertaken in distilled water by means of the potentiometric method using a pH-meter N-5170 TELEKO and a complex electrode ESAgP-309W EUROSENSOR. The pH meter was calibrated using buffer solutions of pH 7 and pH 9. Measurements were performed after 3 h following the preparation of the suspension of 1:2.5 (*w*/*v*) dust/water.

In the next stage of research, samples were subjected to mineralogical and chemical analysis by the application of the scanning electron microscopy (SEM) technique, together with energy dispersive spectrometry (EDS). The SEM analysis was performed using an environmental scanning electron microscope Philips XL 30 ESEM/TMP with an analytic EDS unit (EDAX detector, Sapphire type). A primary beam with an accelerating voltage of 15 keV was applied. Using this analytical method, images recording both morphology and size of magnetic particles separated from the dust samples were obtained. Additionally, the chemical micro-analysis with the use of a CAMECA SX100 electron microprobe was conducted on selected samples. For SEM and microprobe analysis the TMPs (technogenic magnetic particles) fraction was enriched by magnetic separation. The separation was held in isopropanol, using an ultrasonic washer, which simplified the separation of individual grains.

Samples were also subjected to mineralogical analysis using the X-ray diffraction technique by powder method. Mineralogical composition of the samples was determined by means of standards, using a Philips PW 3710 X-ray diffractometer and X’PERT computer program. In the X-ray analysis, the Rietveld method was applied to quantify the contribution of each phase to the examined magnetic concentrates.

The selected samples were subjected to thermomagnetic analysis. Measurements were performed using the AGICO KLY-4S susceptibility bridge, equipped with a CS-3 heating chamber, employing a temperature range from room temperature to 700 °C, in ambient air, with a heating rate of 8.5 °C/min.

## Results

Magnetic susceptibility of different kinds of industrial dust is considerably variable, but is generally very high. Compared with other types of analyzed industrial dust, fly ash originating from hard coal burning is characterized by the highest value of magnetic susceptibility. The average values of *χ* obtained for ten ash samples from nine Polish power plants burning hard coal and thermal power stations in all cases exceed a value of 1,000 × 10^−8^ m^3^ kg^−1^, reaching the value of 8,500 × 10^−8^ m^3^ kg^−1^ (Table 1). These values not only depend on the origin of burned coal and the total content of iron in coal, but also on combustion conditions.

**Table 1:** Magnetic susceptibility, *χ*, frequency-dependent magnetic susceptibility, χ_fd_, and pH of fly ash, cement, and lime dust samples

Sample no.	Description	*χ* (×10^−8^ m^3^ kg^−1^)	*χ*_fd_ (%)	pH
1.	Dust from hard coal combustion	7,428	2.9	8.8
2.	Dust from hard coal combustion	2,588	1.2	9.0
3.	Dust from hard coal combustion	5,049	1.0	9.0
4.	Dust from hard coal combustion	8,516	2.1	9.2
5.	Dust from hard coal combustion	7,941	2.8	9.9
6.	Dust from hard coal combustion	1,691	1.1	10.0
7.	Dust from hard coal combustion	5,650	2.2	9.1
8.	Dust from hard coal combustion	3,776	3.6	10.3
9	Dust from hard coal combustion	7,364	0.9	9.3
10	Dust from hard coal combustion	4,102	3.5	9.4
11	Dust from lignite combustion	590	1.3	8.8
12	Dust from lignite combustion	552	1.0	9.2
13.	Dust from lignite combustion	552	0.7	9.4
14.	Dust from lignite combustion	582	1.5	8.5
15.	Dust from lignite combustion	548	0.1	8.4
16.	Cement dust—first section of electrostatic precipitator	82	5.4	8.4
17.	Cement dust—second section of electrostatic precipitator	66	4.2	8.8
18.	Cement dust—behind electrostatic precipitator	806	2.2	9.2
19.	Cement dust—behind electrostatic precipitator	97	2.1	11.9
20.	Cement dust—dustfall	233	1.9	7.9
21.	Cement dust—first section of electrostatic precipitator	199	1.9	8.2
22.	Cement dust—second section of electrostatic precipitator	101	2.7	9.4
23.	Cement dust—behind electrostatic precipitator	142	2.1	8.6
24.	Lime plant—chimney	5	–	11.9
25.	Lime plant—chimney electrostatic precipitator	3	–	7.0
26.	Lime plant—worm	2	–	11.5
27.	Lime plant—worm electrostatic precipitator	1	–	8.0

A characteristic feature of all fly ash samples from hard coal burning is the low value of frequency-dependent magnetic susceptibility (*χ*_fd_). This parameter did not exceed the value of 4 % in any of the tested samples. Such low values of *χ*_fd_ means an almost total absence of superparamagnetic grains (magnetic particles < 0.02 μm) within the magnetic fraction. This is a factor which allows ferrimagnetic grains of anthropogenic origin, originating from fly ash fallout that usually occurs as a multidomain size, to be distinguished from natural ferrimagnetic grains of biogenic origin (produced by magnetotactic bacteria), or from pedogenic grains formed as a result of precipitation of iron minerals from soil solutions. This parameter is therefore helpful in detecting the presence of industrial dust in the topsoil.

Fly ash originating from lignite combustion has lower *χ* values. In five tested samples the values ranged between 548 and 590 × 10^−8^ m^3^ kg^−1^. The *χ*_fd_ parameter reached a maximum value of 1.5 % (Table 1).

The average *χ* values for cement and lime dust varied in a very wide range from 1 × 10^−8^ m^3^ kg^−1^ to more than 800 × 10^−8^ m^3^ kg^−1^ (Table 1). The lowest *χ* values are characteristic of paramagnetic minerals and reveal a complete lack of ferrimagnetic iron oxides in the mineralogical composition of this dust. Such a wide variation is caused by the various additives used during cement production. These materials are often iron-bearing wastes from metallurgy, power plants, ore processing etc. Metallurgical wastes and fly ashes from hard coal combustion are of the greatest importance in the process of magnetic enrichment of cement dust (Gołuchowska 2001).

Almost all tested samples were characterized by pH values greater than 8.0, and so were alkaline.

**Fig. 1:**
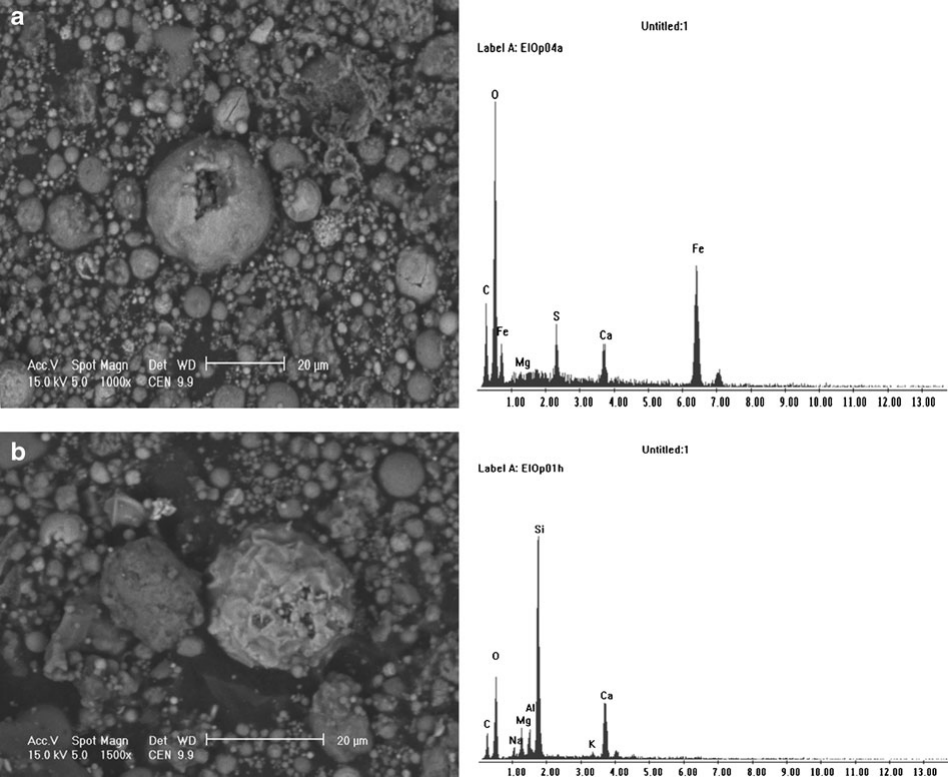
SEM micrographs of fly ash from hard coal burning power plant with EDS spectra: **a** accumulation of hollow particles with iron oxide composition, **b** quartz particle

Observations of fly ash particles from the combustion of hard coal with SEM technique showed that a significant share, both in magnetic and silicate phases, have spherical forms of various sizes from several to dozens of micrometers, called magnetic spherules (Puffer et al. 1980). Some have a smooth surface, and others a corrugated or even skeletal morphology. Magnetic iron oxides frequently occur in the form of incrustation on the surface of mullite, amorphous silica, or aluminosilicate. Other spherules consist entirely of a ferrimagnetic shell built of iron oxide (magnetite and maghemite), and their inside is void with visible holes after the exit of the gas which originally filled the spherules (Fig. 1a).

The components of fly ash also include aluminosilicate particles occurring frequently in oval or spherical forms, usually with a smooth surface. Angular forms were rarely observed. The forms of spherical or near-spherical aluminosilicates are typical of coal combustion processes. Silica (SiO_2_) present in fly ash occurs in crystalline form (quartz and mullite) as well as in a glassy phase form. Particles of quartz were observed in the form of spherical and angular grains of 10 to 18 μm diameter (Fig. 1b), containing admixtures of magnesium, aluminum, and calcium. Calcite (CaCO_3_) particles were also observed in the form of angular grains with smooth surfaces, with a size not exceeding 10 μm.

**Fig. 2:**
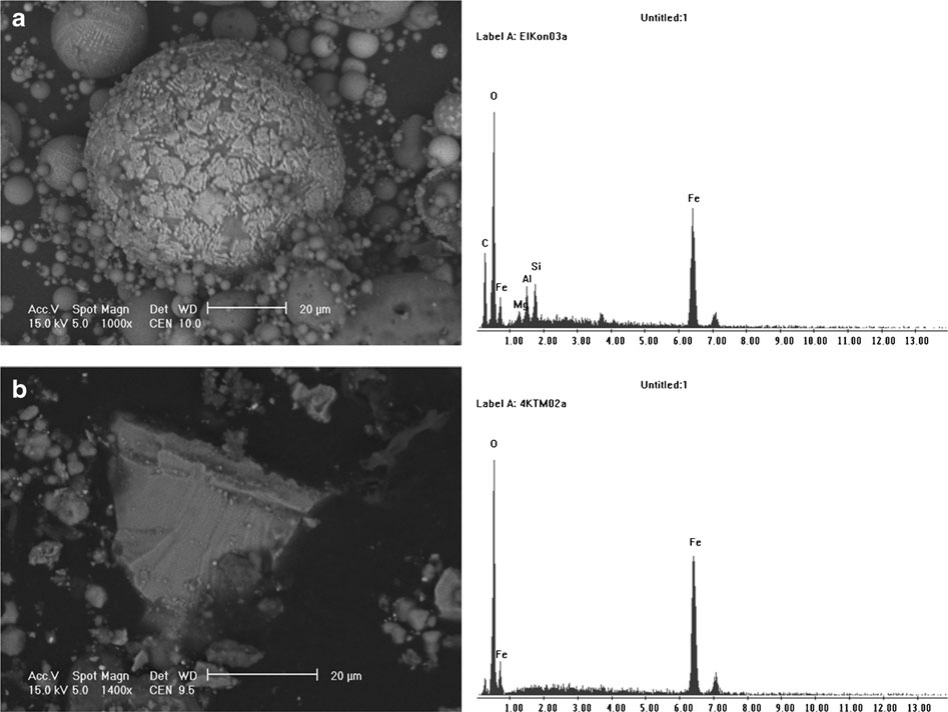
SEM micrographs of fly ash from lignite burning power plant with EDS spectra: **a** incrustation of iron oxide on aluminosilicate spherical particle, **b** angular particle of iron oxide

**Fig. 3:**
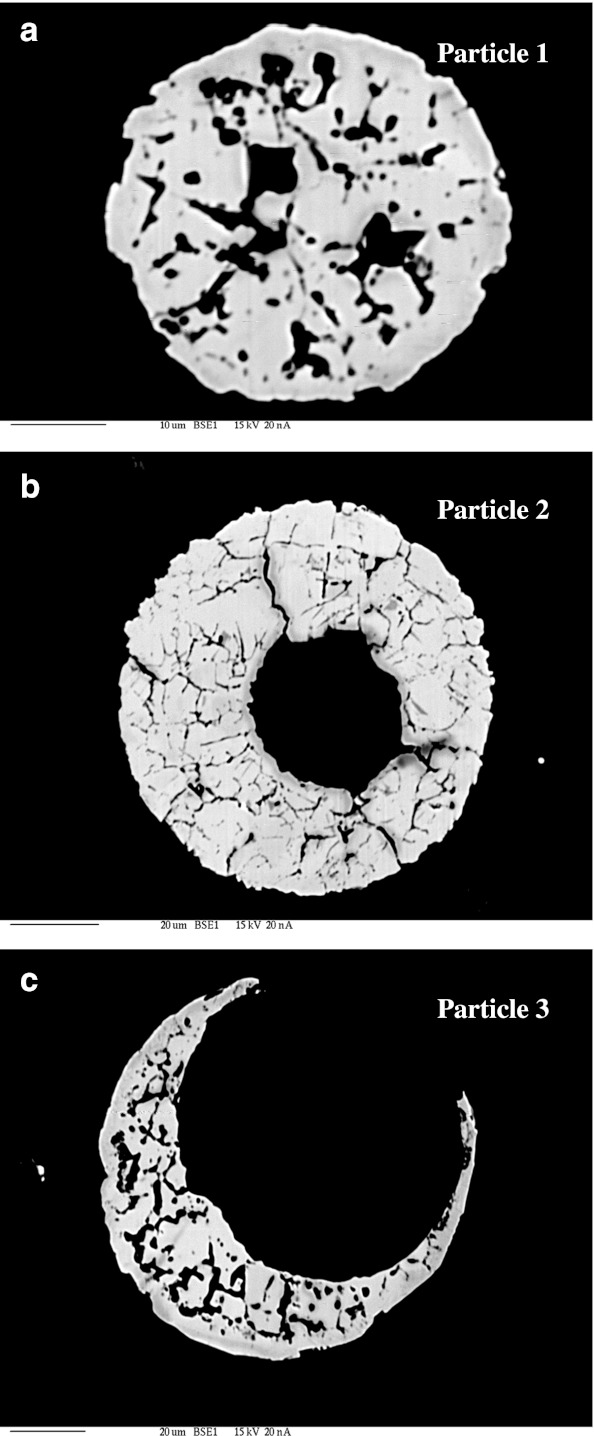
Cross-section through the complete (**a**) and hollow (**b** and **c**) magnetic spherules. Pictures taken with the microprobe on magnetic concentrate extracted from fly ash after lignite burning. Chemical composition of these particles is shown in Table 2

Morphological forms of minerals occurring in ash originating from the combustion of lignite did not differ from the forms of those with an origin in the combustion of hard coal. In samples of fly ash formed after lignite combustion, aluminosilicate particles occurring with the elements barium, potassium, magnesium, and iron were frequently identified. These took spherical forms with smooth surfaces with a size not exceeding 50 μm. The spherules were varied, from forms with completely smooth surfaces to forms with extensive corrugated surfaces. Forms with incrustation of iron oxides (probably hematite) on the silicate core of magnetic spherules were also found (Fig. 2a). The magnetic fraction was also present in the form of angular grains (Fig. 2b). Cross-sections of magnetic particles observed with the microprobe showed that they have a contrast phase composition built of both non-stoichiometric iron oxides of maghemite–magnetite series and hematite coexisting with siliceous glaze, mullite, or other forms of silica and aluminosilicates (Fig. 3, Table 2). The metal oxides were often present in the form of incrustations, and some formed empty crusts with visible holes. These forms were often cracked with a very extensive internal surface which can adsorb heavy metals, as confirmed by stoichiometric analyses (Table 2).

**Table 2:** Stoichiometric analysis of chemical composition of magnetic particles no. 1, 2, and 3 shown in the Fig. 3 occurring in magnetite concentrate extracted from fly ash after lignite combustion

	Particle 1	Particle 2	Particle 3
P_2_O_5_	0.006	0.019	0.010
SiO_2_	0.087	0.000	0.047
SO_2_	0.012	0.000	0.043
TiO_2_	0.031	0.021	0.002
Al_2_O_3_	0.000	0.037	0.113
Fe_2_O_3_	99.731	98.102	100.081
MgO	0.297	0.135	0.582
CaO	0.213	0.041	0.278
MnO	0.000	0.000	0.302
CoO	0.066	0.065	0.038
NiO	0.144	0.032	0.023
CuO	0.000	0.000	0.045
ZnO	0.094	0.000	0.000
CdO	0.048	0.019	0.000
PbO	0.003	0.044	0.000
Na_2_O	0.000	0.011	0.000
Total (%)	100.711	98.526	101.564

**Fig. 4:**
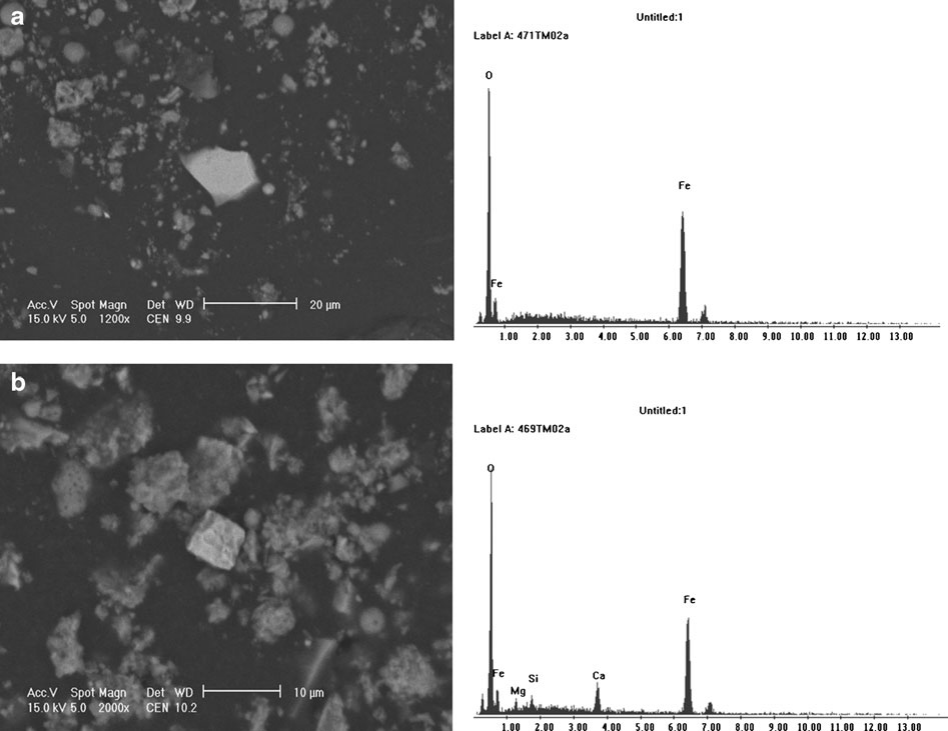
SEM micrographs of the magnetic concentrate extracted from cement dust with EDS spectra: **a** irregular magnetite particle in dust falling into the area of cement plant, **b** octahedral particle of iron oxide (probably calcium ferrite CaFe_3_O_5_) in emitted cement dust collected behind electro-filter

The magnetic fraction of cement dust occurred mostly in the form of angular and octahedral grains of a significantly finer granulation (<20 μm). The oxide forms of iron were commonly found there, in many cases with a considerable admixture of calcium, suggesting calcium ferrites with the chemical composition CaFe_3_O_5_, rather than pure stoichiometric magnetite (Fig. 4).

**Fig. 5:**
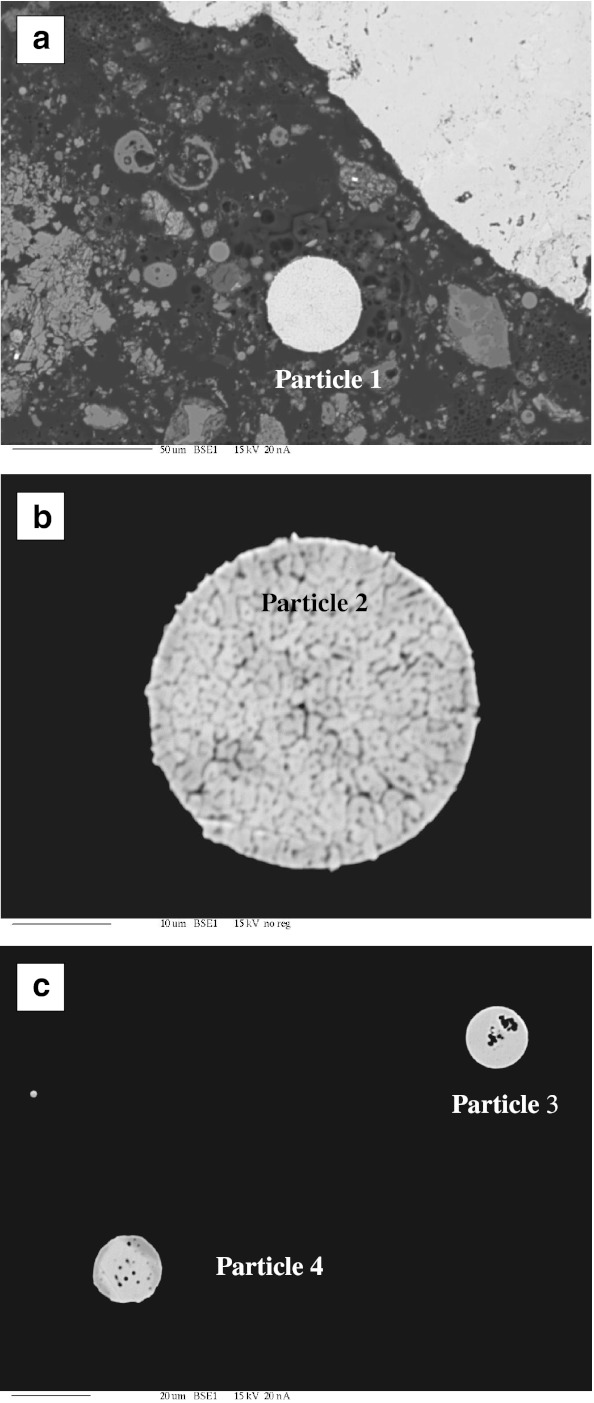
Cross-section through the complete spherules. Pictures taken with microprobe on magnetic concentrate of cement dust falling into the area of cement plant. Chemical composition of these particles is shown in Table 3

Magnetic spherules as well as angular forms were also present. Diphase structure of spherules was often seen on the cross-sections through magnetic spherules visible in the pictures taken by the microprobe (Fig. 5). Their interior is probably close to the magnetite phase, and the outer zone is made up of maghemite (Table 3).

**Table 3:** Stoichiometric analysis of the chemical composition of spherical particles no. 1, 2, 3, and 4, shown in Fig. 5, occurring in magnetic concentrate extracted from cement dust

	Particle 1	Particle 2	Particle 3	Particle 4
P_2_O_5_	0.031	0.000	0.057	0.002
SiO_2_	1.115	1.501	1.867	0.061
SO_2_	0.018	0.022	0.116	0.014
TiO_2_	0.020	0.033	0.677	0.031
Al_2_O_3_	1.330	1.339	2.207	0.248
Fe_2_O_3_	97.931	98.167	67.791	100.353
MgO	0.378	0.391	0.604	0.430
CaO	0.225	0.314	22.251	0.073
MnO	0.093	0.081	0.243	0.074
CoO	0.000	0.059	0.038	0.028
NiO	0.003	0.047	0.033	0.000
CuO	0.000	0.000	0.000	0.000
ZnO	0.000	0.020	0.000	0.000
CdO	0.000	0.000	0.017	0.000
PbO	0.111	0.000	0.064	0.000
Na_2_O	0.038	0.016	0.041	0.000
Total (%)	101.332	101.990	95.506	101.320

**Fig. 6:**
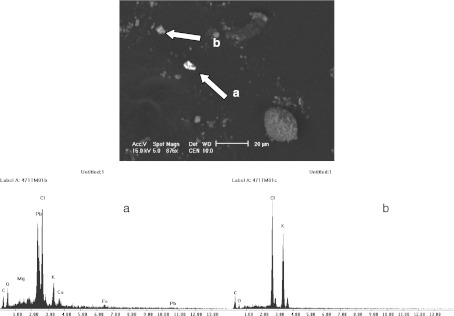
SEM micrographs of lead chloride (**a**) and sylvinite (**b**) with EDS spectra–cement dustfall

In the non-magnetic phase, aluminosilicate particles were present with admixtures of iron, sodium, potassium, and magnesium, probably derived from the application of silica-bearing additives, mainly quartz sand and waste silica, to the production of clinker in the tested cement plants. They occurred as spherical and irregular, rough or smooth forms of 10 to 20 μm in size. Particles of calcite were also observed in cement dust, probably derived from the use of raw materials for clinker production. Molecules of sylvinite in the shape of irregular forms with a diameter of several microns were also noted, as were slightly larger particles of lead chloride occurring in the forms of angular grains with smooth surfaces and dimensions from 3 to 10 μm (Fig. 6).

**Fig. 7:**
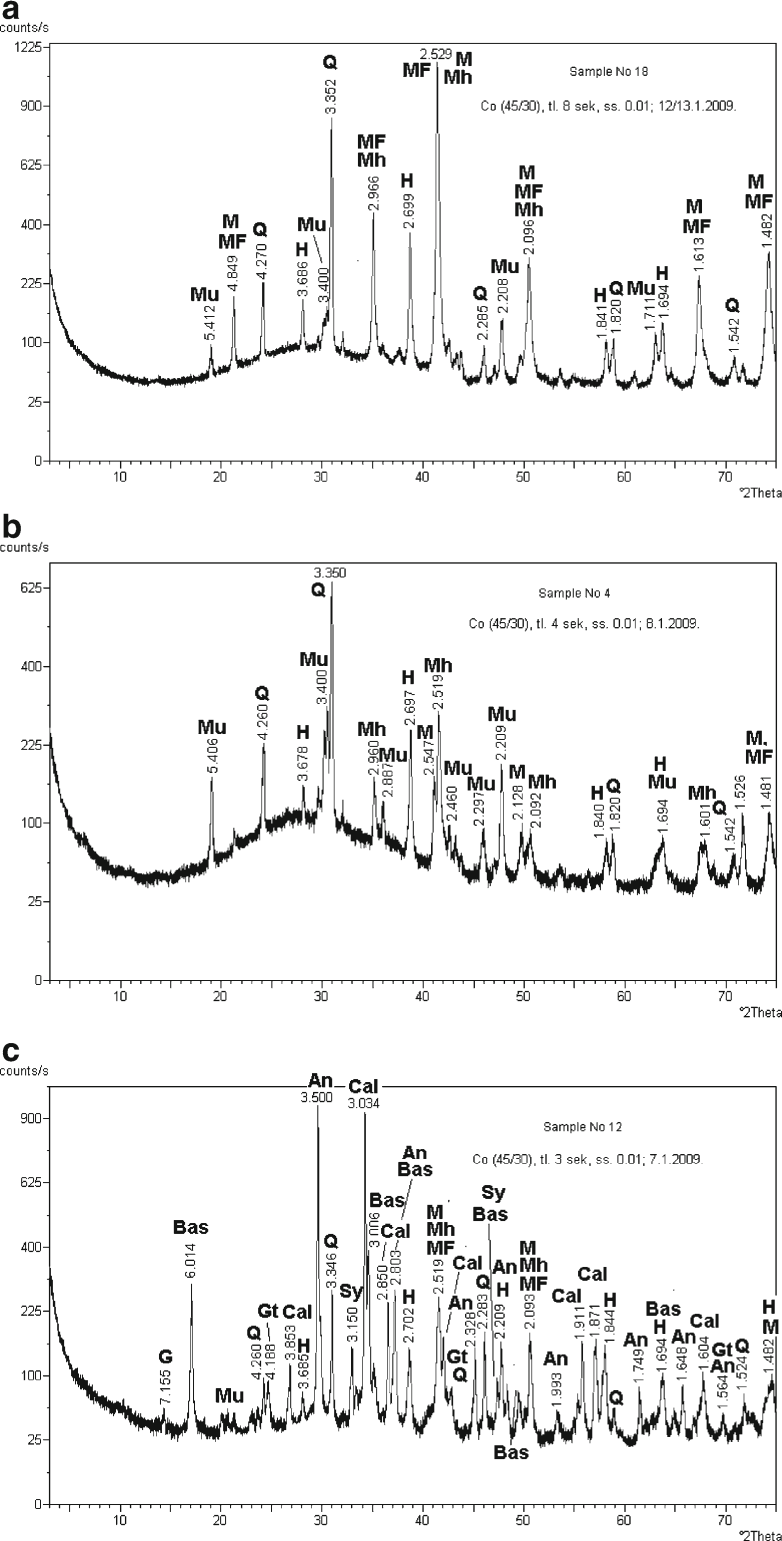
X-ray diffraction pattern of the following samples: **a** fly ash from hard coal burning, **b** fly ash from lignite burning, **c** cement dust. *Mh* maghemite, *MF* magnesioferrite, *M* magnetite, *H* hematite, *Mu* mullite, *Q* quartz, *Gt* goethite, *An* anhydrite, *Cal* calcium, *Bas* bassanite, *G* gypsum (Magiera et al. 2011)

Former studies determined diffraction patterns of the magnetite concentrates extracted from samples of ash and cement dust as well as thermomagnetic analyses of ash samples (Magiera et al. 2011). In the case of ash from hard coal burning, these studies show the dominance of three ferrimagnetic minerals: magnetite, magnesioferrite, and maghemite. Hematite occurs here in much smaller quantities (Fig. 7a). The amount of quartz and mullite, which are physically linked (sintered), was also significant.

Maghemite and hematite are dominant in the magnetic minerals in ash formed from lignite combustion, while strong ferrimagnetic minerals such as magnetite and magnesioferrite occur as subordinate components (Fig. 7b); this explains the much lower magnetic susceptibility of this ash in relation to the ash from hard coal combustion.

The analyses of X-ray diffraction patterns show that—in terms of mineralogical composition—the magnetic fraction in cement dust samples is much more diverse than in the other kinds of tested dust. The dominant magnetic minerals here are magnetite, maghemite, and also the forms of ferrites with a significant content of calcium. Other than these dominant magnetic minerals, hematite and goethite are also present within the magnetic phase. Ferrimagnetic minerals in magnetic concentrates are accompanied by a large amount of diamagnetic minerals, such as anhydrite, calcite, bassanite, gypsum, and quartz (Fig. 7c).

**Fig. 8:**
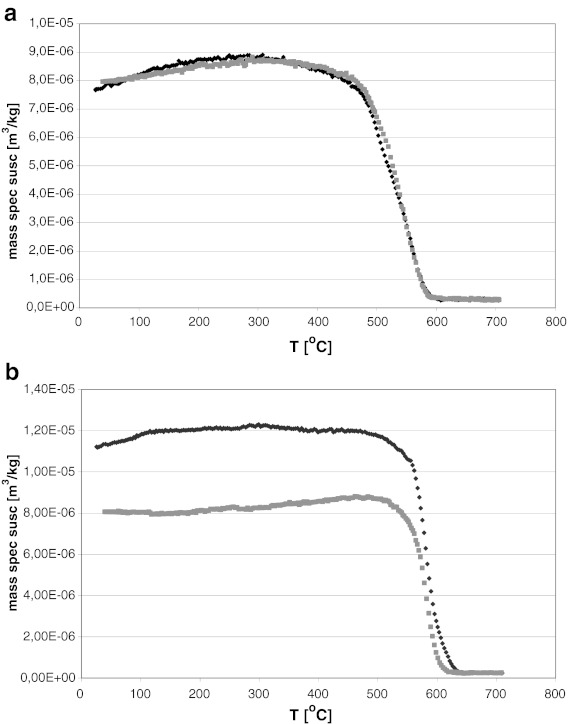
Thermomagnetic curves of two samples of fly ash emitted by power plant: **a** hard coal burning, **b** lignite burning. *Black* heating line, *grey* cooling line (Magiera et al. 2011)

Thermomagnetic analyses performed on fly ash samples showed that the dominant magnetic phase in dust from the combustion of hard coal is magnetite with a specific Curie temperature of ∼580 °C. During heating of the sample to a temperature of 700 °C, no formation of any other magnetic minerals was noted, and the curves for heating and cooling were almost identical (Fig. 8a). In the case of ash after the burning of lignite, thermomagnetic analyses show that the Curie point is shifted well above a temperature of 600 °C, suggesting the likely presence of significant quantities of maghemite (Curie point ca. 640 °C) or hematite (the Neel temperature of pure hematite is 675 °C). The magnetic susceptibility of the cooling curve was lower than the initial magnetic susceptibility, suggesting that certain parts of magnetite or maghemite were transformed into weakly magnetic forms (Fig. 8b).

**Fig. 9:**
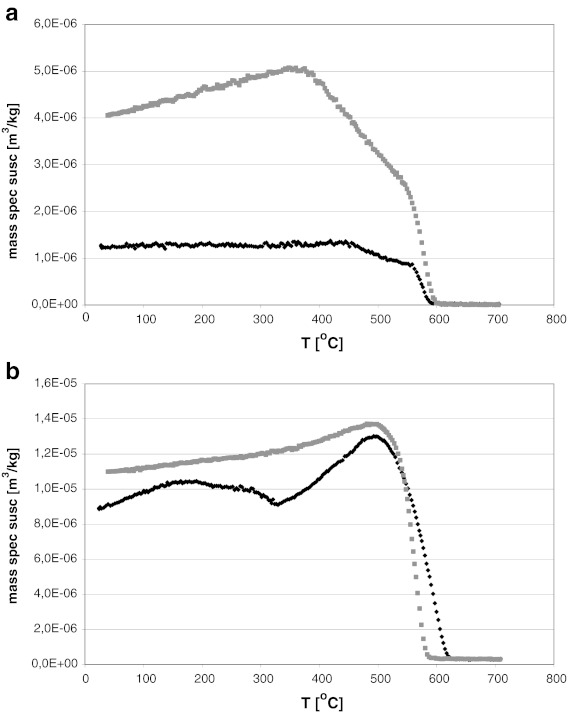
Thermomagnetic curves of two cement dust samples collected from chimney electofilters (**a**—zone I, **b**—zone II). *Black*—heating line, *grey*—cooling line

Thermomagnetic curves of cement dust measured during this study (only those with a value of *χ* above 50 × 10^−8^ m^3^ kg^−1^ were subjected to thermomagnetic studies) were more diverse, but in all tested samples the dominance of magnetite as the main magnetic mineral was clearly marked, occurring most often within a fairly wide range of particle size. In most tested samples, the presence of hematite also cannot be excluded (Fig. 9). Sometimes there were also rises in magnetic susceptibility at temperatures above 300 °C, suggesting the presence of sulfides. In some samples of cement dust, two magnetic phases clearly appeared on the cooling curve (at a temperature of 450 and 550 °C). For the majority of cement dust samples, the cooling curves were above the heating curves, and the magnetic susceptibility after thermal treatment was two to four times higher than at the beginning. This suggests the transition of weakly magnetic mineral phases into ferrimagnetic forms, which presumably means that, during the heating of the sample, secondary magnetite occurs.

A comparison of all mineral forms of alkaline dust identified in the tested samples is presented in Table 4. The basic components of dust samples from hard coal combustion in power plants are amorphous substance (glassy phase; ∼40 % vol.) and mullite (∼15 %), with a slightly lower amount of magnesioferrite/magnetite with hematite (Σ ∼ 12 %), quartz (∼10 %), MgO in the form of periclase (∼8 %), probably the Ca-Mg silicate (smectite), lime CaO, and a trace content of anhydrite.

**Table 4:** Mineral phases identified in alkaline dust samples

Mineralogical composition of alkaline dust samples		
Power plant dusts—from hard coal combustion	Power plant dusts—from lignite combustion	Cement dust
Mullite 3Al_2_O_3_ × 2SiO_2_	Anhydrite CaSO_4_	Bassanite CaSO_4_ × 0.5H_2_O
Magnetite FeFe_2_O_4_	Hematite α-Fe_2_O_3_	Anhydrite CaSO_4_
Magnesioferrite MgFe_2_O_4_	Maghemite β- Fe_2_O_3_	Calcite CaCO_3_
Hematite α-Fe_2_O_3_	Magnetite FeFe_2_O_4_	Hematite α-Fe_2_O_3_
Quartz SiO_2_	Lime CaO	Magnetite FeFe_2_O_4_
Periclase MgO	Periclase MgO	Ca-ferrite CaFe_3_O_5_
Lime CaO	Portlandite Ca[OH]_2_	Goethite α- FeOOH – β-FeOOH
Smectite	Quartz SiO_2_	Anthropogenic calcium sulfate CaSO_4_
M_*x*_(Al_1.67_ Mg_0.33_)[Si_4_O_10_](OH)_2_	Ca_3_Al_6_O_12_ × CaSO_4_ phase	Sylvinite KCl
Anhydrite CaSO_4_	Merwinite Ca_3_Mg[SiO_4_]_2_	Ammoniojarosite (NH_4_)Fe_3_[SO_4_]_2_(OH)_6_
	Akermanite Ca_2_Mg[Si_2_O_7_]–gehlenite Ca_2_Al[(Al,Si)_2_O_7_]	Quartz SiO_2_
	Bassanite CaSO_4_ × 0.5H_2_O	Kaolinite Al_4_[Si_4_O_10_](OH)_8_
	Mullite 3Al_2_O_3_ × 2SiO_2_	Illite (K_9_H_3_O^+^)Al_2_[AlSi_3_O_10_](OH)_2_
	Calcium silicate CaO × SiO_2_	Ettringite Ca_6_Al_2_[SO_4_(OH)_4_]_3_ × 24 H_2_O

The phase composition of dust samples after lignite burning in power plants include anhydrite (∼25 % vol.), maghemite with hematite and magnetite (Σ ∼ 20 %), CaO (∼12 %), MgO (∼10 %), portlandite, quartz, the Ca_3_Al_6_O_12_·CaSO_4_ phase, merwinite, the intermediate member of the akermanite-gehlenite series, and the admixture of bassanite and mullite. Difficult-to-determine calcium silicate is also present (reflex ∼ 9.3–9.4 Å).

Three main substances occurred in the mineral composition of cement dust: anhydrite with bassanite (Σ ∼ 32 %), and calcite (∼28 %). Smaller amounts were found of: hematite with magnetite and goethite (Σ ∼ 15 %), sylvinite and/or CaSO_4_, ammoniojarosite, quartz, kaolinite, illite, and ettringite.

## Discussion

Recognition of the morphological and mineralogical features of alkaline dust, especially the magnetic fraction contained therein, is essential to reveal the magnetic properties of iron minerals as indicators of soil pollution caused by the deposition of industrial dust, including fly ash, onto the soil. The performed analyses confirmed the presence of a considerable amount of technogenic magnetic particles in alkaline dust. The easily measurable value of magnetic susceptibility is directly proportional to the content of these particles in the measured sample and can be used to estimate the anthropogenic dust content in topsoil. The tested dust showed different values of *χ*, mostly dependent on the type of coal, the temperature and conditions of combustion in power boilers, the type of raw materials, additives and fuels used for cement and lime production, the size of their consumption, as well as the manufacturing method for cement and lime.

The highest value of magnetic susceptibility was shown by the fly ash samples from the combustion of hard coal. This is caused by the high temperature of the combustion resulting in a high content of ferrimagnetic minerals. The major source of iron in hard coal is framboidal pyrite. At the temperatures of coal combustion, the conversion of weakly magnetic sulfides (pyrite and marcasite) into the ferrimagnetic forms of iron oxides (magnetite and maghemite) takes place (Mitchell and Gluskoter 1976; Lauf et al. 1982). The creation of maghemite was observed within the temperature range of 800–1,400 °C and magnetite 1,300–1,600 °C. Some authors also suggest that, at a temperature approximately 550 °C, the dissociation of pyrite occurs, resulting in conversion to troilite via ferrimagnetic pyrrhotite (Gryglewicz et al. 1996; Gupta et al. 2007).

The ease with which pyrite undergoes dissociation and oxidation makes it a particularly significant substrate in the high-temperature processes of the formation of ferrimagnetic minerals. Pyrite is one of the most important iron minerals present in coal, with a content reaching 15 %.

Fly ash from the combustion of lignite showed slightly lower but also high values of magnetic susceptibility, with a maximum average value of *χ* reaching 590 × 10^−8^ m^3^ kg^−1^. The results of qualitative analysis and scanning electron microscopy confirmed the high content of ferrimagnetic minerals in dusts from power plants. In fly ash from hard coal combustion, the content of magnesioferrite/magnetite phase together with hematite was ∼12 %, and from lignite combustion the contribution of magnetite or maghemite with hematite was ∼20 %. In the case of fly ash from hard coal, ferrimagnetic magnesioferrite/magnetite minerals were predominant among TMPs, whereas in the case of ash from lignite combustion a considerable amount of maghemite (which is also ferrimagnetic, but with a lower spontaneous magnetization) and/or antiferromagnetic hematite was observed. Iron oxides in the analyzed fly ash samples occurred in the specific morphological form of magnetic spherules with a grain size of 20–50 μm, but there were also a considerable number of grains smaller than 10 μm.

Cement dust was much more diverse, both magnetically and mineralogically. The X-ray phase analysis and SEM analysis also confirmed the participation of magnetic particles in cement dust. The results of the study showed that the values of *χ* of cement dust samples are highly variable. For some kinds of dust it was above 800 × 10^−8^ m^3^ kg^−1^, providing a high content of ferrimagnetic minerals and, in other cases, mainly in the dust from lime plants, it had a value of 1 or 2 × 10^−8^ m^3^ kg^−1^, which is at the level of diamagnetic or paramagnetic minerals, confirming a marginal content of iron minerals in the samples.

As proved during earlier studies (Gołuchowska and Strzyszcz 1999; Gołuchowska 2001), the sources of magnetite in cement dust are raw materials and additives used in cement production and ash from the combustion of hard coal in cement kilns. Iron-bearing additives have the greatest impact on the magnetic susceptibility of cement dust and should be considered the most dangerous to the environment.

The lowest values of magnetic susceptibility are characteristic of alkaline dust from lime plants, which contains mostly diamagnetic and paramagnetic substances. This may be related to the fact that—compared with cement production— lime production technology does not use corrective additives, the so-called low raw materials such as marl, clay etc., as well as coal to fire lime kilns.

The research into TMPs in atmospheric dust collected in Upper Silesia (Southern Poland) has shown that most of the magnetic particles have a spherical shape, characteristic of particles produced in the processes of solid fuel (hard coal) combustion (Strzyszcz 1993; Magiera et al. 2002; Magiera et al. 2010). These are called magnetic spherules and their size varies from a few to several dozen micrometers. Some have a smooth surface, others are corrugated, and some are fused with the silicate phase (mainly the mullite or glassy phase).

The internal structure of the spherules is twofold. Some spheres, having a smaller diameter, are solid and consist of a magnetic core consisting of non-stoichiometric spinel of a magnetite–maghemite series (Fe_3-*x*_Me_*x*_O_4_), or a mineral with the structure of ferrite (MeFe_2_O_4_) (Hullet et al. 1980; Strzyszcz et al. 1996). They are usually surrounded by a thin silicate, aluminosilicate, or calcareous coating. The magnetic spherules of the second type are hollow, and their shell is mainly composed of magnetite or an intermediate phase of magnetite–maghemite. There are visible holes on the surface of the latter, being the outlets of the gas. These were formed during rapid solidification of iron oxides and silica at high temperature, as evidenced by the significant amounts of silicate glassy phase and mullite.

The results of the study showed that technogenic magnetic particles, being a carrier of magnetic signals in atmospheric dust, often also contain significant amounts of elements, including heavy metals (Mn, Zn, and Ni; Tables 2 and 3). They can be bound either in a spinel crystalline structure, or by the forces of surface adsorption. In the latter case, after deposition on the soil surface, particularly in forest areas where soils are usually acidic, they may be relatively easily released into the environment, posing a potential ecological threat.

The analysis of the results of SEM-EDS studies showed that there are differences in the kinds of metals associated with magnetic particles at the different sampling sites. In the area of Dąbrowa Górnicza–Łosień (Upper Silesia, Southern Poland), situated in the direct range of the influence of the “Katowice” Steelworks, and especially in the area of Siemianowice–Michałkowice, in close proximity to “Jedność” Steelworks, and a few other old steelworks of the central part of the Upper Silesian Industrial Region, a relationship between magnetic particles and nickel and chromium was found, these being the specific metals associated with the iron and steel industry. A greater quantity of angular particles was also observed in the atmospheric dust in this area. Their presence in soil organic levels had previously been reported in the areas of ferrous metallurgy, particularly near the old steel plants (Magiera et al. 2002; 2008). Atmospheric dust samples collected in these areas clearly showed the highest χ value. This was not only a result of the largest accumulation of ferrimagnetic iron oxides, but also the presence of metallic iron (α Fe), which occurred in the form of angular particles. Metallic iron is the strongest ferromagnet and its magnetic susceptibility is approximately 400 times higher than the magnetic susceptibility of the stoichiometric magnetite (Thompson and Oldfield 1986).

Magnetic particles present in dust falling onto the soil surface may cause an increase in the magnetic susceptibility of the upper soil horizons (Kusza and Strzyszcz 2005). According to Strzyszcz (1993), magnetite content in soils in the immediate vicinity of industrial plants is high. Furthermore, the increase in magnetic susceptibility of the soil covered by alkaline dust emissions is a result of the enrichment of soil in iron oxides, which may act as carriers of heavy metals. Zawadzki et al. (2004) confirms a high correlation between the rise in magnetic susceptibility and the increased content of heavy metals in industrial dust. In the case of cement dust, high correlation coefficients with magnetic susceptibility were observed for the following metals: iron, manganese, lead, and cadmium and, in the case of fly ash, for iron, manganese, zinc, and lead.

Taking into account the significant correlation between industrial emissions and increased magnetic susceptibility of the soil, and the fact that magnetic particles can be carriers of heavy metals, methods for magnetic measurement should be considered as particularly useful in monitoring the state of the soil environment.

## Conclusions

- Alkaline dust released into the atmosphere from coal combustion and cement production processes and deposited in the topsoil have pH values between 8 and 12. They contain technogenic magnetic particles that can be used as an easily measurable tracer of this kind of pollution.
- In fly ash from coal burning power plants, TMPs occur in very characteristic spherical forms of various sizes from several to dozens of micrometers, the so-called magnetic spherules. Ferrimagnetic iron oxides (magnetite and maghemite) frequently form incrustation on the surface of mullite, amorphous silica, or aluminosilicate particles. Other spherules consist entirely of a ferrimagnetic shell built of iron oxide, and their inside is void with visible holes after gas escape.
- In fly ash from lignite combustion, maghemite and hematite prevail while strong ferrimagnetic minerals such as magnetite and magnesioferrite occur as a minor fraction, which explains the much lower magnetic susceptibility of this ash in comparison with the ash from hard coal combustion.
- The mineralogical nature of TMPs in cement dust is more diverse. The magnetic fraction of cement dust occurs mostly in the form of angular and octahedral grains of a significantly finer granulation (<20 μm). In many cases, ferrimagnetic minerals occur here with a considerable admixture of calcium. Sometimes they form calcium ferrites (CaFe_3_O_5_), rather than pure stoichiometric magnetite or maghemite. Iron-bearing additives have the greatest impact on the magnetic susceptibility of cement dust, and should be considered the most dangerous to the environment.
- Stoichiometric analyses confirmed the presence of heavy metals such as Pb, Mn, Cd, and Zn connected with TMPs, which are carriers of a magnetic signal in atmospheric dust, and therefore in some cases their presence in topsoil detected by magnetic measurement can be treated as an indicator of inorganic soil contamination.
